# Enhanced Recovery After Surgery Protocols in Pediatric Surgery: A Narrative Review of Current Evidence

**DOI:** 10.7759/cureus.100748

**Published:** 2026-01-04

**Authors:** Sol Angie Rincon Mora

**Affiliations:** 1 Department of Surgery, Fundación Universitaria Sanitas, Bogotá, COL

**Keywords:** enhanced recovery after surgery, eras protocols, multidisciplinary care, pain management, pediatric surgery, perioperative care, postoperative recovery

## Abstract

Enhanced Recovery After Surgery (ERAS) protocols have been successfully implemented in adult surgical practice, demonstrating reductions in postoperative complications, the length of hospital stay, and healthcare costs. In pediatric surgery, however, the adoption of ERAS principles has progressed more slowly due to age-related physiological variability, the heterogeneity of surgical procedures, and the limited availability of high-quality evidence in certain pediatric populations.

This narrative review aims to summarize and critically evaluate the current evidence on the implementation of ERAS protocols in pediatric surgery, with a focus on core principles, clinical outcomes, implementation challenges, and future directions. A narrative review of the literature was conducted using PubMed and Scopus databases. Articles published between 2010 and 2025 were identified using keywords related to enhanced recovery, pediatric surgery, and perioperative care. Original studies, reviews, and clinical guidelines involving pediatric surgical patients were included, while studies exclusively involving adult populations, editorials, and conference abstracts without full text were excluded.

Available evidence suggests that the implementation of ERAS protocols in pediatric surgery is associated with shorter hospital stays, improved postoperative pain control, the earlier resumption of oral intake and mobilization, and high levels of patient and family satisfaction, without an increase in postoperative complications. Most of the published evidence originates from pediatric colorectal, abdominal, and urologic surgeries. However, challenges remain regarding protocol standardization, multidisciplinary team adherence, and the adaptation of ERAS pathways to different age groups, particularly neonates and infants.

ERAS protocols represent a promising strategy for optimizing perioperative care in pediatric surgery. Although current evidence supports their safety and effectiveness in selected procedures, further prospective and multicenter studies are needed to develop age-specific protocols and expand ERAS implementation across pediatric surgical subspecialties.

## Introduction and background

The development of Enhanced Recovery After Surgery (ERAS) emerged from a growing recognition that postoperative morbidity is largely driven by the physiological stress response to surgery. Early perioperative studies in adult patients demonstrated that coordinated, multimodal strategies, combining optimized analgesia, minimally invasive techniques, and early mobilization and nutrition, could significantly reduce the postoperative length of stay and accelerate functional recovery after major abdominal procedures. These observations challenged the traditional view that pain, ileus, fatigue, and delayed recovery were unavoidable consequences of surgery and laid the conceptual foundation for standardized, evidence-based perioperative care pathways that prioritize recovery rather than prolonged convalescence [1].

This approach evolved within a broader shift toward quality- and safety-focused surgical care, driven by the increasing awareness of the central role of the physiological stress response in postoperative morbidity. Traditional perioperative practices, such as prolonged fasting, opioid-based analgesia, excessive fluid administration, and delayed mobilization, have been shown to exacerbate neuroendocrine activation, inflammation, postoperative pain, and gastrointestinal dysmotility. In contrast, multimodal perioperative pathways aimed at attenuating surgical stress have demonstrated consistent improvements in postoperative recovery, including reductions in pain, postoperative ileus, and hospital length of stay across adult surgical disciplines. These principles were later formalized through guideline-driven ERAS pathways, most notably by the ERAS Society. More recently, these concepts have begun to be applied to pediatric surgical care, where emerging evidence suggests potential benefits in clinical outcomes and patient and family satisfaction, although implementation remains heterogeneous and relatively recent [2,3].

ERAS is a multimodal perioperative care approach aimed at optimizing patient recovery by reducing surgical stress and improving physiological function through coordinated interventions across the preoperative, intraoperative, and postoperative phases of care. While ERAS protocols are well established in adult surgical practice, their application in pediatric populations presents unique challenges. Children represent a highly heterogeneous group with age-dependent physiological characteristics, distinct metabolic requirements, limited physiological reserves, and neurodevelopmental factors that influence pain perception, stress responses, and postoperative cooperation. In addition, caregiver involvement plays a central role in perioperative decision-making and adherence to recovery pathways, necessitating age-specific adaptations and limiting the direct extrapolation of adult-based protocols to pediatric surgical care [4].

In pediatric surgery, the application of ERAS principles has gained increasing attention, with several studies reporting favorable outcomes such as the reduced length of hospital stay, improved pain control, and the earlier resumption of oral intake and mobilization, particularly in colorectal, abdominal, and urologic procedures. However, the adoption of ERAS protocols has not been uniform across pediatric surgical subspecialties, and the available evidence remains fragmented, with considerable variability in protocol composition, implementation strategies, and outcome reporting. These limitations, together with barriers related to multidisciplinary acceptance and adherence, highlight the need to consolidate and critically synthesize existing evidence to guide the development of standardized, age-appropriate ERAS pathways in pediatric surgery. Therefore, the aim of this narrative review is to summarize and critically analyze the current evidence on ERAS implementation in pediatric surgery, focusing on fundamental principles, clinical outcomes, implementation challenges, and future directions [4,5].

## Review

Application of ERAS in pediatric surgery

The core components of ERAS protocols in pediatric surgery typically include multimodal analgesia, the early initiation of enteral nutrition, goal-directed fluid therapy, early mobilization, and standardized perioperative care pathways. These elements are designed to reduce the surgical stress response, facilitate the faster recovery of bowel function, and shorten the hospital length of stay while maintaining patient safety [4].

ERAS protocols have been most extensively implemented in pediatric colorectal surgery, which represents the subspecialty with the most robust evidence supporting enhanced recovery pathways in children. In this setting, standardized perioperative care pathways have been compared to conventional management and have primarily been applied to major abdominal procedures. These pathways emphasize procedure-specific adaptations of core ERAS principles, with particular attention to analgesia, nutrition, perioperative fluid management, and strategies aimed at accelerating the recovery of bowel function. Importantly, these pathways are adapted to pediatric physiological characteristics and perioperative needs, reflecting the complexity of translating ERAS principles to younger populations [3,5,6].

Beyond colorectal surgery, the application of ERAS protocols has also been explored in other pediatric surgical subspecialties, particularly pediatric urology. In urologic procedures, ERAS pathways are generally composed of multiple predefined elements, and their implementation is often evaluated based on adherence to individual components rather than the application of a uniform pathway. Not all ERAS elements are applicable to every procedure, highlighting the importance of procedure-specific and age-appropriate adaptations when implementing enhanced recovery strategies in pediatric patients [3,5,7].

In addition, recent systematic reviews have evaluated the application of ERAS principles in pediatric minimally invasive surgery, demonstrating favorable postoperative outcomes and supporting the broader applicability of enhanced recovery pathways across pediatric surgical subspecialties.

Overall, the application of ERAS in pediatric surgery remains concentrated in selected subspecialties, with considerable variability in protocol composition and implementation across institutions. This heterogeneity reflects differences in surgical complexity, patient age, and local practice patterns, as well as the evolving nature of ERAS adoption in pediatric care. Successful implementation frequently depends on close multidisciplinary collaboration and the active involvement of patients and families to ensure adherence and safety within pediatric ERAS pathways [5,8].

Intraoperative management within pediatric ERAS pathways

Intraoperative management constitutes a central component of pediatric ERAS pathways and plays a key role in attenuating the surgical stress response and facilitating postoperative recovery. Core intraoperative strategies include the use of standardized anesthetic techniques, multimodal opioid-sparing analgesia, and optimized intraoperative fluid administration. These measures aim to minimize neuroendocrine activation, reduce postoperative pain and gastrointestinal dysmotility, and promote early functional recovery.

In pediatric colorectal surgery, the implementation of a pediatric-specific enhanced recovery protocol incorporating intraoperative optimization has been shown to be both feasible and safe. The use of multimodal analgesic strategies and more judicious fluid administration has been associated with reduced postoperative narcotic requirements and lower intraoperative fluid volumes, contributing to shorter hospital stays without an increase in postoperative complications or readmissions. These findings underscore the importance of tailored intraoperative ERAS strategies as a critical determinant of successful enhanced recovery in pediatric surgical populations [6].

Clinical outcomes associated with ERAS

The implementation of Enhanced Recovery After Surgery (ERAS) protocols in pediatric surgical practice has been associated with favorable postoperative outcomes across multiple surgical subspecialties. Overall, available evidence suggests that ERAS pathways promote more efficient postoperative recovery without an increased risk of postoperative complications when appropriately adapted to pediatric patients [9,10].

One of the most consistently reported benefits of ERAS implementation in pediatric surgery is a reduction in the hospital length of stay. Studies involving pediatric colorectal, abdominal, and urologic procedures have demonstrated earlier discharge among patients managed under standardized ERAS protocols compared to conventional perioperative care [9,11]. In pediatric colorectal surgery, both George et al. [12] and Purcell et al. [13] reported significant reductions in the length of stay following ERAS adoption, without an associated increase in complications. More recent evidence indicates that these improvements are sustained over time, with persistent reductions in the length of stay observed following long-term ERAS implementation [14].

Improved postoperative pain management represents another key outcome associated with ERAS pathways. The use of multimodal analgesic strategies has been shown to reduce postoperative opioid consumption in pediatric patients while maintaining adequate analgesia [9,11]. Consistent with these findings, George et al. observed lower postoperative opioid use and reduced pain scores throughout hospitalization following ERAS implementation [12].

The early initiation of oral feeding and the accelerated recovery of bowel function have also been frequently reported within ERAS frameworks. Strategies aimed at minimizing perioperative fasting and promoting early enteral nutrition support gastrointestinal recovery and may reduce the risk of postoperative ileus in pediatric patients [9,10].

Regarding postoperative complications and readmission rates, current evidence indicates that ERAS protocols can be safely applied in selected pediatric surgical procedures without compromising patient safety. Multiple studies, including those by George et al. [12], Purcell et al. [13], and Luo et al. [14], have reported no increase in postoperative complications or readmissions following ERAS implementation.

Challenges and limitations

Although interest in Enhanced Recovery After Surgery (ERAS) protocols in pediatric surgery continues to grow, several challenges limit their broad and consistent implementation. A major concern is the wide variation in how ERAS pathways are applied across institutions and pediatric surgical subspecialties. Differences in protocol structure, perioperative practices, and adherence levels contribute to inconsistent implementation and hinder meaningful comparisons between studies [15,16].

In addition to developmental heterogeneity, pediatric perioperative care presents unique clinical challenges, including pain management, perioperative anxiety, and respiratory complications, that differ substantially from those encountered in adult patients and may impede the uniform implementation of standardized recovery protocols [17,18].

The diversity of the pediatric population further complicates ERAS standardization. Pediatric patients range from neonates to adolescents and exhibit marked physiological, developmental, and nutritional differences. These factors often require tailored, age-specific, and procedure-specific adaptations of ERAS pathways, which may reduce the feasibility of applying uniform protocols across all pediatric surgical populations [15,16].

Limitations in the current body of evidence also restrict definitive conclusions regarding ERAS effectiveness in pediatric surgery. Many published studies are observational, single-center investigations with limited sample sizes. The lack of large, prospective, and multicenter studies weakens the overall strength of the evidence and increases the risk of bias. Additionally, variations in study design and outcome definitions make it challenging to synthesize results across the existing literature. Narrative reviews and meta-analyses consistently highlight the heterogeneous nature of the available evidence and underscore the need for more robust and standardized research [16,18].

Practical implementation barriers represent another significant challenge. The limited awareness of ERAS principles, reluctance to modify established perioperative routines, and the requirement for coordinated multidisciplinary teamwork may impede protocol adoption. Effective implementation often depends on institutional support, structured education, and ongoing quality monitoring, resources that may not be consistently available in all clinical settings [15,16].

To address these challenges, recent consensus statements in pediatric perioperative care have emphasized the importance of standardized yet flexible, age-specific, and multidisciplinary strategies to enhance perioperative safety and recovery and to support more consistent ERAS implementation across institutions [15,16].

Future directions

Advancing the application of Enhanced Recovery After Surgery (ERAS) in pediatric surgery will require the generation of more robust and methodologically sound evidence. Future research should prioritize prospective, multicenter study designs to better evaluate the safety, feasibility, and effectiveness of ERAS protocols across diverse pediatric age groups and surgical subspecialties, addressing current limitations related to small sample sizes and single-center investigations [15,18].

Another important priority is the development of standardized yet flexible ERAS pathways tailored to patient age and procedure type. The marked physiological and developmental differences among neonates, infants, children, and adolescents necessitate age-specific perioperative strategies rather than uniform recovery protocols in pediatric surgical care [15,16].

Improving the translation of ERAS principles into routine clinical practice will also be critical. Evidence from pediatric ERAS reviews and implementation-focused literature highlights the importance of multidisciplinary education, institutional commitment, and continuous audit and feedback systems to ensure protocol adherence and long-term sustainability [18,19]. In pediatric settings, the active engagement of caregivers throughout the perioperative period may further support safe implementation and improve overall satisfaction [15].

In addition, experience from ERAS Society guidelines in adult surgery underscores that enhanced recovery should be regarded as a dynamic, audit-driven process rather than a static protocol. High levels of protocol adherence, structured multidisciplinary collaboration, and continuous quality improvement have been identified as key determinants of sustained clinical benefit. These implementation principles may provide a useful conceptual framework to guide the future development and optimization of pediatric ERAS pathways [20,21].

Finally, future studies should expand beyond traditional clinical outcomes to include patient-centered and caregiver-reported measures, such as the quality of life, functional recovery, and satisfaction. The adoption of standardized outcome measures and implementation metrics will be essential to enable meaningful comparisons across studies and to support ongoing optimization of ERAS protocols in pediatric surgery [18].

## Conclusions

In summary, ERAS protocols represent a valuable and evolving approach to optimizing perioperative care in pediatric surgery. Available evidence indicates that, when appropriately adapted to pediatric populations, ERAS pathways are associated with improved postoperative recovery, including the reduced length of hospital stay, improved pain control, decreased opioid use, and the earlier return of gastrointestinal function, without an increase in postoperative complications.

Despite these encouraging findings, the implementation of ERAS in pediatric surgery remains limited by heterogeneity in patient populations, protocol design, and study methodology, as well as by institutional and multidisciplinary barriers to adoption. Future progress will depend on the development of age- and procedure-specific ERAS pathways, the generation of high-quality multicenter evidence, and the incorporation of standardized and patient-centered outcome measures. Continued multidisciplinary collaboration and implementation-focused research will be essential to support the safe and consistent integration of ERAS protocols into pediatric surgical practice.
